# Potential Hazard to Human Health from Exposure to Fragments of Lead Bullets and Shot in the Tissues of Game Animals

**DOI:** 10.1371/journal.pone.0010315

**Published:** 2010-04-26

**Authors:** Deborah J. Pain, Ruth L. Cromie, Julia Newth, Martin J. Brown, Eric Crutcher, Pippa Hardman, Louise Hurst, Rafael Mateo, Andrew A. Meharg, Annette C. Moran, Andrea Raab, Mark A. Taggart, Rhys E. Green

**Affiliations:** 1 Wildfowl & Wetlands Trust, Slimbridge, Gloucestershire, United Kingdom; 2 Residues Surveillance Unit, Veterinary Medicines Directorate, Addlestone, Surrey, United Kingdom; 3 Instituto de Investigación en Recursos Cinegéticos, IREC (CSIC-UCLM-JCCM), Ciudad Real, Spain; 4 Institute of Biological Environmental Sciences, University of Aberdeen, Aberdeen, United Kingdom; 5 Royal Society for the Protection of Birds, Sandy, Bedfordshire, United Kingdom; 6 Conservation Science Group, Department of Zoology, University of Cambridge, Cambridge, United Kingdom; University of Lethbridge, Canada

## Abstract

**Background:**

Lead is highly toxic to animals. Humans eating game killed using lead ammunition generally avoid swallowing shot or bullets and dietary lead exposure from this source has been considered low. Recent evidence illustrates that lead bullets fragment on impact, leaving small lead particles widely distributed in game tissues. Our paper asks whether lead gunshot pellets also fragment upon impact, and whether lead derived from spent gunshot and bullets in the tissues of game animals could pose a threat to human health.

**Methodology/Principal Findings:**

Wild-shot gamebirds (6 species) obtained in the UK were X-rayed to determine the number of shot and shot fragments present, and cooked using typical methods. Shot were then removed to simulate realistic practice before consumption, and lead concentrations determined. Data from the Veterinary Medicines Directorate Statutory Surveillance Programme documenting lead levels in raw tissues of wild gamebirds and deer, without shot being removed, are also presented. Gamebirds containing ≥5 shot had high tissue lead concentrations, but some with fewer or no shot also had high lead concentrations, confirming X-ray results indicating that small lead fragments remain in the flesh of birds even when the shot exits the body. A high proportion of samples from both surveys had lead concentrations exceeding the European Union Maximum Level of 100 ppb w.w. (0.1 mg kg^−1^ w.w.) for meat from bovine animals, sheep, pigs and poultry (no level is set for game meat), some by several orders of magnitude. High, but feasible, levels of consumption of some species could result in the current FAO/WHO Provisional Weekly Tolerable Intake of lead being exceeded.

**Conclusions/Significance:**

The potential health hazard from lead ingested in the meat of game animals may be larger than previous risk assessments indicated, especially for vulnerable groups, such as children, and those consuming large amounts of game.

## Introduction

Lead is highly toxic affecting most body systems. Blood lead thresholds of concern have decreased 6-fold in the last 50 years as knowledge of the effects of lead has increased [1]–[5]. Whilst lead is toxic to all age groups, foetuses and young children are the most vulnerable. Intake and gastrointestinal absorption of lead is higher in children and the effects of lead more pronounced on developing systems [6], [7]. Recent research from the UK and USA has documented effects on cognitive function, educational attainment and IQ in children at levels well below the current action threshold of 10 µg dL^−1^ blood lead [8]–[10]. These findings have stimulated recent calls by health experts for a halving of current action thresholds for blood lead [10], [11]. UK and other European Union governments have committed to numerous international resolutions and declarations, and are bound by regulations aimed at minimising the impacts of lead on human health and the environment, particularly for high-risk groups, vulnerable groups such as children, and to reducing levels of lead in food. A primary route of exposure of humans to lead is through ingestion of contaminated foodstuffs. Commission Regulation 1881/2006 [12] sets maximum levels (MLs) for certain contaminants in food, including lead. However, whilst a wide range of foodstuffs derived from domesticated and wild organisms are listed within 1881/2006 and have MLs set for lead, wild game, which is shot with lead ammunition in most European countries, is not included. Other regulatory commitments include European Community Regulation EC 1907/2006 [13] on chemicals and their safe use (the REACH Regulations).

Many scavenging and predatory birds and mammals ingest lead gunshot or bullets along with their food, including deer viscera discarded by hunters, unretrieved quarry, and prey animals with ingested gunshot in the digestive tract or which have been shot but survive carrying lead pellets in their flesh. Lead poisoning from ammunition sources is a well established cause of mortality in many birds of prey globally [14]. Meat from game animals shot using lead ammunition is also a potential source of dietary exposure in humans, but was previously believed to pose a minimal hazard because most of the mass of the projectile(s) remained in one large piece, either passing through the carcass or being removed during food preparation or at the table. However, recent radiographic studies show that the meat of deer and wild boar shot with lead bullets contained bullet fragments which, in many cases, were small, numerous and widely dispersed relative to the wound canal [15]–[17]. Lead from fragments of bullets used to shoot deer has recently been shown to be bioavailable. Ingestion of venison containing small bullet fragments, remaining after standard meat preparation by butchers, resulted in significantly increased blood lead concentrations in pigs [16]. A recent survey in North Dakota showed that blood lead levels were correlated with the frequency and amount of meat consumed from wild deer and other game animals [18]. Concern has also been raised about lead contamination from the meat of game animals killed using lead gunshot, as well as bullets. Lead concentrations in breast muscle from some seabirds killed for food using lead shot in Greenland were considered sufficiently high for sustained consumption to be potentially hazardous to human health, even though all visible shot were removed from the tissues before analysis [19]. Bjerregaard and colleagues [20] found blood lead concentrations in Greenlanders to be correlated with reported levels of consumption of seabirds killed using gunshot and blood lead levels in adult Inuit people in arctic Canada were positively correlated with the quantity of hunted waterfowl in the diet [21]. Taken together, these studies indicate that, even though the level of bioavailability of metallic lead from gunshot and bullet fragments is likely to differ from and could be less than that of biologically incorporated lead or lead salts [22], it nonetheless appears to cause elevation of blood lead.

Wild-shot game can form an important component of the diets of certain social and economic groups across the European Union, including leisure and subsistence hunters, and those with an ethical preference for consuming wild animals. In the UK, most of the game animals killed are terrestrial gamebirds and deer which are legally and normally shot with lead gunshot or bullets. Tens of millions of gamebirds are reared and released for shooting each year. Wild-shot game is marketed and sold at a variety of outlets, including supermarkets, and a campaign ‘Game to Eat’ has actively promoted game consumption in the UK over the last eight years, with consumption reported to have increased in response and forecast to increase further [23]. Previous assessments of the risk posed to consumers in the UK by dietary lead derived from spent ammunition have assessed the risk as low for meat from gamebirds and non-negligible for wild ducks [24]–[26]. In this paper, we investigate whether lead derived from spent gunshot and bullets in the tissues of game animals in the UK could pose a threat to human health and report that the potential health hazard is larger than previously supposed.

## Materials and Methods

### Sample Collection and Preparation

Wild-shot pheasant (*Phasianus colchicus*), red-legged partridge (*Alectoris rufa*), woodpigeon (*Columba palumbus*), red grouse (*Lagopus lagopus*), woodcock (*Scolopax rusticola*) and mallard (*Anas platyrhynchos*) were purchased from supermarkets, game dealers/butchers, and directly from gamebird shoots in England, Scotland and Wales by staff of the Wildfowl & Wetlands Trust (WWT). All carcasses were made oven-ready (feathers, viscera and heads were discarded from all birds) and X-rayed using a PLH Medical K6 Electronic portable machine at 70 kilovoltage potential and 10 milliamps. A focal distance of 75cm was used with Mediphot X-ray HDC-UVB high contrast, blue sensitive radiographic film (30.0cm×40.0cm). Exposure time was 0.8 seconds. The plates were processed using an X'Ograph Nodark automatic processor using Devalex M (Champion) and fixed using Fixer Plus (Champion). Numbers of shot and large radio-dense fragments were recorded, with fragment size being estimated to the nearest 10% of a whole pellet by mass. Tiny (generally <5% pellet) radio-dense particles visible on the X-ray were also recorded on a four-point ordinal scale, as follows: 1 = 0 to 5 fragments, 2 = 6 to 10 fragments, 3 = 11 to 15 fragments and 4 = more than 15 fragments. For analysis of the number of visible shot per individual, a count was made of the number of pellets and fragments judged to comprise at least half of the mass of a whole pellet (e.g. an apparently intact pellet plus a fragment judged to comprise 0.6 of a pellet would be counted as 2). For analysis of lead concentration in relation to the quantity of spent ammunition visible on X-rays of the carcass, we used the number of pellet equivalents, with all fragments greater than 10% of an intact pellet being summed (the example given above would be scored as 1.6 pellets).

For each species, the sample of birds was divided into two groups. An attempt was made to match the groups in terms of the distribution of the numbers of embedded shot visible on X-rays. The birds were then prepared to simulate realistic exposure of humans to lead by consumption of cooked meat. Typical cooking recipes for each species were identified through an internet search (Text S1). Two cooking recipes were selected for each species: one likely to result in approximately neutral pH, e.g. roasting or stewing with a water or cream-based sauce, and one likely to be more acidic, e.g. involving wine or cider. We call these “non-acidic” and “acidic” recipes, although the pH of the liquid around the bird was not recorded. One group of birds was cooked using each recipe. Chicken breast muscle (from a supermarket) was used as a control for each species and cooking recipe. Once cooked, any apparently whole gunshot or large fragments of gunshot that could have been detected by the consumer (i.e. ≥half a shotgun pellet), were removed by dissection and stored, and the cooked meat was separated from the skeleton. Meat and sauce samples were homogenised separately using a hand blender, weighed and oven dried at 20°C to constant weight. All equipment was thoroughly cleaned between samples by washing in hot water with detergent using plastic brushes, rinsing in 1% Decon, then rinsing in distilled water. A 5g aliquot of dried material from each meat and sauce sample was sent for analysis. Randomly selected aliquots from dried homogenised samples from 10 each of partridges and pheasants (five cooked using an acidic and five a non-acidic recipe) were also sent for analysis to determine the heterogeneity of lead concentrations among aliquots from the same homogenised sample. All samples were stored in plastic tubes sealed with Parafilm in containers with desiccants to ensure that they remained dry.

We also analysed data on lead concentrations in game animals obtained from the statutory surveillance programme of the Veterinary Medicines Directorate (VMD). Analytical work undertaken under this programme was not designed for risk assessment purposes, and the data were collected and analysed in a different way from those in the WWT study. Previously, only summary data from the VMD programme have been made publicly available, and the raw VMD data provides additional pertinent and valuable information to the WWT study. Consequently we used both sets of data, although they have, of necessity, been treated in a somewhat different manner, as described below.

As part of the VMD statutory surveillance programme, wild pheasants and partridges (whole birds from game shoots) and samples of wild venison (deer species unknown, from slaughterhouses) were collected. For gamebirds, one side of the breast and the opposite leg were homogenized. An aliquot of homogenized tissue was analysed for lead analysis. Gunshot pellets, where present, were not removed. Remaining tissue was stored. For venison, sections of muscle were received for analysis. Connective tissue was removed prior to homogenizing.

### Qualititative identification of the composition of gunshot pellets taken from gamebirds

Numbers of radio-dense pellets were counted on X-rays of each carcass in the WWT sample (see above). All pellets were tested to see whether they were attracted by a bar magnet. Magnetic pellets were considered to be steel and not further examined. For the majority of birds where at least three shot were present, the presence/absence of lead in one or two of the shot recovered was determined, as a variety of shot types is available. Where two or fewer shot were recovered, the assay for lead was not performed because it is destructive and it was considered desirable to retain shot for possible isotopic analysis at a later stage. Provisional identification of pellet type was based upon appearance, malleability and melting point. Pellet type was confirmed when melting point results were consistent with the results of qualitative chemical analysis (and where these were not inconsistent with general appearance and malleability). Known pellet types were used as positive controls throughout the analyses.

The colour and form of the non-magnetic pellets were examined. Those with a slight steely or golden reddish tint were suspected to be made from an alloy composed mainly of bismuth. Dark, dull and deformed pellets were suspected to be lead or possibly bismuth alloy. When sanded with fine grain sandpaper and cut with a scalpel blade, those exposing shiny surfaces were considered to be either lead or bismuth alloy. If exposed surfaces appeared slightly golden reddish, they were suspected to be bismuth alloy. Relatively soft pellets were suspected to be lead, harder and more brittle pellets were suspected to be bismuth alloy or another material other than lead.

Fragments cut from pellets were heated for 10 minutes in a partitioned porcelain tray in a muffle furnace at 295°C and 330°C. Lead has a melting point of 327.5°C and bismuth of 271.3°C. Fragments melting at 295°C and forming molten shiny globules upon manipulation were considered to be bismuth alloy. Fragments retaining their shape and remaining hard at 295°C, yet becoming soft at 330°C were considered to be lead.

Pellets were warmed at 90°C and simultaneously shaken on a hot plate in 10ml of 12.5% nitric acid for five minutes, after which 2ml of 10% potassium iodide solution was added. Those forming a bright yellow precipitate (i.e. resembling lead iodide) were considered to contain lead. Those forming a dull amber/dark orange yet clear solution (i.e. resembling bismuth iodide) were considered to be bismuth alloy. Those resulting in any other reaction were classified as unidentified, irrespective of their melting point and other characteristics.

### Measurement of lead concentration

Samples collected by VMD were analysed at the Laboratory of the Government Chemist (LGC), Teddington, Middlesex. Samples collected by WWT were analysed at the Institute of Biological and Environmental Sciences, University of Aberdeen and the Instituto de Investigación en Recursos Cinegéticos (IREC), CSIC, Ciudad Real, Spain.

Both fresh (at LGC) and dried (at IREC and Aberdeen) material was digested using standard procedures. Accurately weighed organic matter (ca. 2.0g fresh material at LGC and ca 0.3g dried material at Aberdeen and IREC) was heated at set temperatures with concentrated Aristar grade nitric acid and hydrogen peroxide until completely digested resulting in a clear straw coloured liquid. The digest was used for analysis.

LGC and Aberdeen measured lead concentrations using inductively coupled plasma - mass spectrometry (ICP-MS). Exact methods are not available from LGC. Aberdeen used the following procedure. Calibration standards were prepared from a 1000 mg L^−1^ certified lead (Pb) stock solution in 2% HNO_3_. Standard concentrations of lead used ranged from 0–100 µg L^−1^. A 5% Rh-solution was prepared in 1% HNO_3_, and used as an internal standard which was introduced into the sample stream via a T-piece. 103Rh, 206Pb and 208Pb data were captured and final results were based on integration of 50 replicate measurements, each made for 10 ms. Standards were re-analysed every 30 or fewer samples, and used to construct an external calibration curve.

IREC analysed samples using graphite furnace atomic absorption spectroscopy (GF-AAS; AAnalyst800 with autosampler AS800 (Perkin Elmer)) for Pb, using 50µg NH_4_H_2_PO_4_ and 3µg Mg(NO_3_)2 as matrix modifiers for each atomisation. Calibration standards were prepared from a 1000 mg L^−1^ certified Pb stock solution in 2% HNO_3_. Standard concentrations used ranged from 0–160 µg Pb/l. Each sample digest was analysed twice and the average reported. A standard and a blank were analysed between every 15 samples during each analytical run to permit regular autozero and calibration reslope.

At least two each of the following Certified Reference Materials (CRM) were used to validate results from Aberdeen and IREC: Dogfish Muscle (DORM2(1)); Lobster hepatopancreas (TORT2, NRC-CNRC National Research Council of Canada), bovine liver and lypophilised pig kidney NR1544, BCR 186 (European Community Bureau of Reference). Recoveries and standard deviations were within acceptable limits.

LGC samples with Pb concentrations that exceeded 150 µg kg^−1^ during the first ‘screening’ analytical run were reanalysed in duplicate for confirmation. The mean of the two concentrations was used. The limit of quantification (LOQ) at LGC was 7 ppb (wet weight). Limits of Detection (LOD) were 31 ppb (dry weight) at IREC and 7.34 ppb (dry weight) at Aberdeen, and we used in our calculations all concentrations above the LOD from these two laboratories.

### Statistical analysis of lead concentrations

We used the raw data to calculate crude observed proportions of samples exceeding the thresholds 100, 1000 and 10000 ppb by wet weight, which are equivalent to 0.1, 1.0 and 10 mg kg^−1^ or ppm. 100 ppb wet weight is the EU (1881/2006) ML permitted in bovine animals, sheep, pigs and poultry (excluding offal). No level has been set for game [12]. For the VMD data, values for samples with concentrations below 300 ppb were not reported for the period 2001–2003, so the observed proportion exceeding 100 ppb is given for the period 2004–2008, during which all concentrations above the LOQ (7 ppb) were available. For the other thresholds, VMD data for the whole period 2001–2008 were used and the two sample sizes given are for the restricted and whole periods.

We also fitted lognormal distributions by a maximum-likelihood method for left-censored distributions implemented in the NONLIN module of SYSTAT 5.0. This was necessary because results from some samples fell below the limits of detection or quantification (LOD or LOQ) of the analytical method or, in the case of the VMD data, values below a certain threshold were not reported (see above). For the WWT study, LOD by dry weight varied between laboratories, so the value appropriate to each sample was used and then converted to a LOD by wet weight by multiplying it by the dry weight fraction for each sample. For the VMD data, the LOQ by wet weight was used for left-censoring for data from 2004–2008, when concentrations determined for screening samples with low values were available. However, for VMD data for 2001–2003, only concentrations above 300 ppb wet weight were available, so this was used as the left-censoring threshold for those years. Initially we fitted separate lognormal distributions to the data from each species-cooking method combination, but we also fitted models to data pooled across cooking methods for each species and for data pooled across cooking methods and species. The statistical significance of differences among cooking methods and species was assessed by comparing residual deviances from the fitted models using likelihood-ratio tests.

We recognize that the WWT study gave rise to concentration estimates from samples that were small in mass relative to the quantity of meat from the bird from which they were derived. The meat of birds killed with lead shot would be expected to contain fragments of metallic lead, even after homogenization. If acidic cooking methods dissolve more of the lead from small fragments, this variation among aliquots would be expected to be smaller for acidic than non-acidic cooking methods. The presence or absence of such particles from the small aliquots taken for analysis would be expected to give rise to variation in lead concentration among aliquots taken from the meat of the same bird. If it is ignored, this variation might inflate the variance and give rise to higher estimated proportions of samples with high lead concentrations than would have been obtained if all of the meat from the bird had been analysed. To allow for this, we estimated variation in concentration among aliquots using five aliquots of meat from each of ten partridges and ten pheasants, half of which were cooked by acidic methods and half by non-acidic methods. Data from one partridge could not be used because all the concentrations fell below the LOD. We fitted lognormal distributions by the method described above in which we assumed a mean concentration specific to each individual bird and a within-bird, among-aliquot variance which was assumed to be the same for all birds within each cooking method category. There was no correlation between the logs of variance and mean for least squares estimates of these parameters calculated for the 17 individuals with no values below the LOD (*r* = 0.059). The estimates of within-bird, among-aliquot variance were 0.893 for acidic cooking and 1.074 for non-acidic cooking. Although this difference is in the expected direction, it was not statistically significant (likelihood-ratio test, χ^2^ = 0.54, d.f. = 1, *P* = 0.541). Hence, the best estimate of within-bird variance was taken to be the value from the fitted model in which cooking method was ignored, 0.991.

We adjusted the observed variation in lead concentration among birds to allow for within-bird variation as follows. We first fitted a lognormal model to the data for each species for the meat component only, excluding samples from sauce for those birds cooked with sauce. For each sample, we then calculated a standardized concentration of lead in the meat (log-transformed value minus the fitted geometric mean for the species, divided by the standard deviation from the fitted model). We then subtracted the within-bird variance from the among-bird variance and took the square root of this as the best estimate of the among-bird standard deviation after adjustment for within-bird variation. We then multiplied the standardized deviation in concentration in the meat for each bird by this adjusted standard deviation and added the species-specific geometric mean concentration in meat. These adjusted values were then combined with the concentrations measured for sauce, as described previously, to give whole bird lead concentrations for meat and sauce combined. A lognormal distribution was then fitted to these adjusted values.

We fitted an asymptotic function to relate the concentration of lead per unit weight of cooked meat in gamebirds from the WWT sample to the number of gunshot pellets detected on X-rays. Concentration was taken to be given by 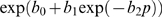, where *p* is the number of pellets and the *b* are constants. The residual standard deviation of the normal variation of log concentration about the geometric mean was taken to be related to pellet number as 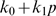. Models were fitted using a maximum-likelihood method in the NONLIN module of SYSTAT 5.0. The model described above was simplified by deleting the effects of pellet number on concentration and on its standard deviation and using likelihood-ratio tests to assess the significance of the resulting changes in residual deviance. We also extended this model to include the effect of the ordinal scale *q* representing the number of small radio-dense fragments visible on the X-ray. In this model, the lead concentration per unit mass of cooked meat was assumed to be given by 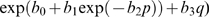. Likelihood-ratio deletion tests were performed to test the effect of small fragment score when the effect of shot and large fragments was included in the model and *vice versa*.

For the purpose of calculating weekly dietary exposure to lead from meals of game meat we calculated the concentration of lead in the whole meal (meat and sauce) relative to the wet weight of the meat component only, having first adjusted the lead concentration in the meat for variation among aliquots as described above. A lognormal distribution was then fitted to these values. The arithmetic mean concentration of lead, relative to the weight of meat in the meal, was then estimated from the estimated mean *m* and standard deviation *s* of the natural logs of the concentrations as 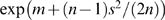.

## Results

### Number of shotgun pellets and small radio-dense fragments found in gamebirds in the WWT sample

The mean number of whole pellets, or large parts of pellets, that were visible on the X-ray images of the WWT sample of gamebirds was 2.17 pellets per bird (Table 1). Over a third of birds (35%) contained no whole pellets or large fragments. There was significant variation among species in the mean number of pellets per bird (Poisson anova; *F_5,114_* = 3.22, *P* = 0.009), with species' means ranging from 0.95 per shot bird^−1^ for woodpigeons to 3.32 shot bird^−1^ for pheasants. There was a significant positive correlation between the mean number of shot detected per bird and the mean body weight of the species (Spearman rank correlation coefficient *r_S_* = 0.886, *P* = 0.05). Mean body weights were taken from a standard ornithological text [27].

**Table 1:** Numbers of gamebirds recorded with various numbers of shot detectable on X-rays.

	Number of birds						
Number of shot	Red grouse	Mallard	Partridge	Pheasant	Woodpigeon	Woodcock	All species
0	3	4	8	10	9	8	42
1	5	2	6	3	7	4	27
2	2	4	3	0	3	3	15
3	5	1	4	0	1	0	11
4	2	1	0	2	1	0	6
5	0	4	2	2	0	0	8
6	1	0	1	0	0	1	3
7	0	0	1	3	0	0	4
8	0	0	1	0	0	0	1
9	0	0	0	0	0	0	0
10	0	0	0	0	0	0	0
11	1	0	0	0	0	0	1
12	1	0	0	0	0	0	1
13	0	0	0	1	0	0	1
14	0	0	0	0	0	0	0
15	0	0	0	0	0	0	0
16	0	0	0	0	0	0	0
17	0	0	0	0	0	0	0
18	0	0	0	1	0	0	1
19	0	0	0	0	0	0	0
20	0	0	0	0	0	0	0
***N***	20	16	26	22	21	16	121
**Mean shot per bird**	3.05	2.31	2.12	3.32	0.95	1.00	2.17

Each whole shot or fragment estimated to be at least half the size of a whole shot was counted.

**Figure 1:**
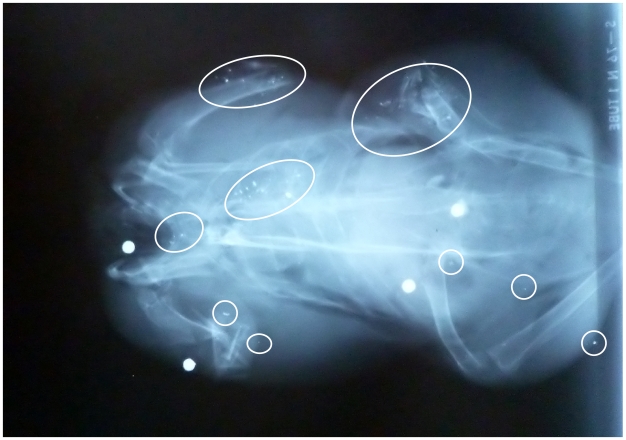
X-ray of a woodpigeon illustrating four gunshot and numerous small radio-dense fragments. Radio-dense fragments may trace the passage of shot through the bird; some fragments are close to bone suggesting fragmentation on impact, others are not.

Small radio-dense particles, presumed to be metallic fragments derived from shotgun pellets, were observed on X-rays of 76% of birds (Table 2: species range 65–85%). The majority of fragments found were very tiny (i.e. less than about a tenth of a shot in size) and both too small and too scattered to be detected or removed by a consumer (e.g., Figure 1). The proportion of birds with shotgun pellets, small fragments or both visible was 87%. The majority (60%) of birds with no shotgun pellets visible on the X-ray had small radio-dense fragments. The small radio-dense particles sometimes appeared to follow the track taken by a shotgun pellet during passage through a bird, were sometimes clustered around bone (Figure 1), but sometimes appeared to be scattered throughout the bird.

**Table 2:** Numbers of gamebirds recorded with various numbers of small radio-dense fragments detectable on X-rays.

	Number of birds						
Small fragments	Red grouse	Mallard	Partridge	Pheasant	Woodpigeon	Woodcock	All species
0	3	5	9	4	4	4	29
1–5	9	6	8	13	12	2	50
6–10	3	3	6	2	4	4	22
11–15	4	1	2	0	1	3	11
>15	1	1	1	3	0	3	9

### Qualitative identification of the composition of recovered gunshot pellets

Recovered shot from all birds were tested for magnetism. Magnetic shot, assumed to be steel, were recovered from two mallard. One bird contained two magnetic shot and the other contained one magnetic and two non-magnetic shot. Other tests were applied to shot taken from 35 birds with more than two shot available for testing. Pellets from 32 (91.4%) birds had the appearance of lead, melted at 330°C and were confirmed as lead by the precipitation of lead iodide on chemical analysis. One bird contained a pellet that had the appearance of lead and melted at 330°C, but resulted in a deep burgundy rather than a yellow precipitate on chemical analysis, and was classified as of unidentified composition. Pellets from the remaining two birds had the appearance of bismuth alloy, melted at 290°C, and produced clear solutions of varying intensity on chemical analysis. These were considered to be free of lead and suspected to be bismuth alloy.

### Concentration of lead in chicken and game

**Figure 2:**
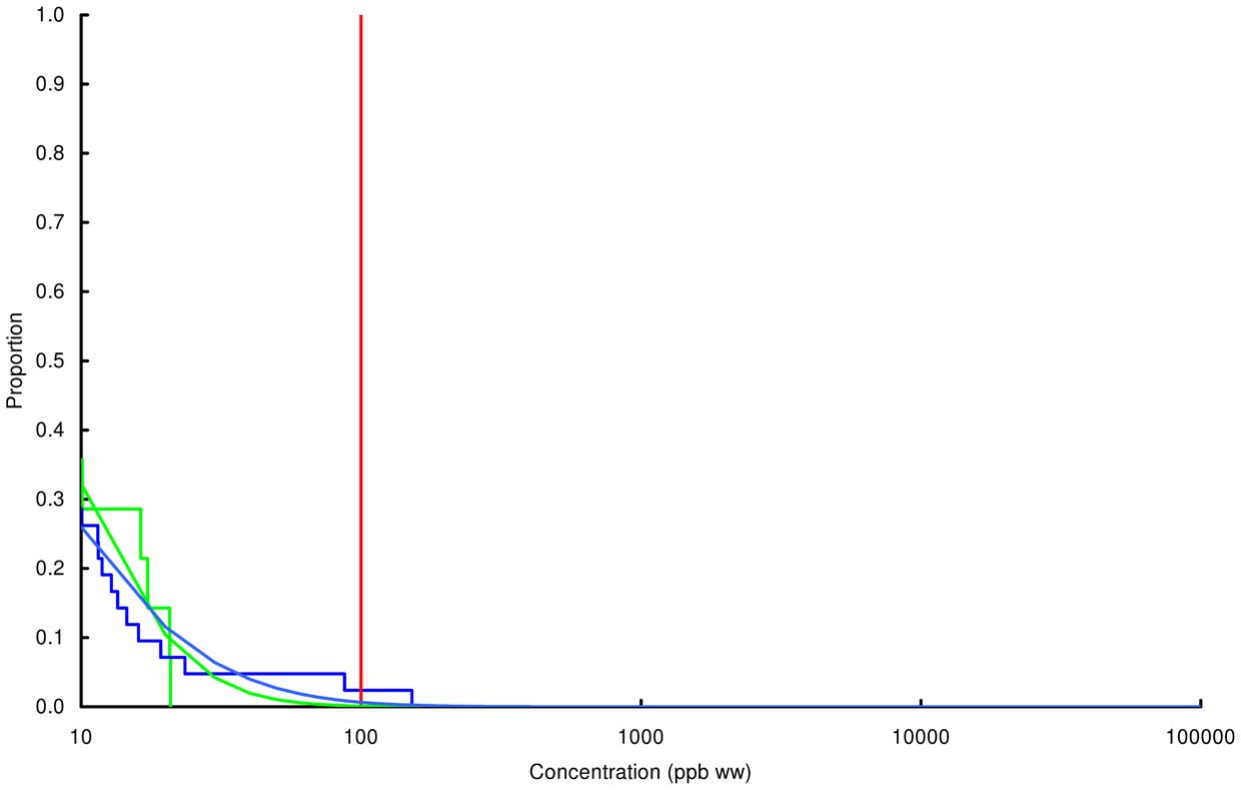
Exceedance distribution of lead concentrations in chicken. Observed (stepped line) and fitted (curve) exceedance distribution of lead concentrations (ppb wet weight) in chicken cooked using acidic (green) and non-acidic (blue) methods. The curves represent lognormal distributions fitted to all the data, including those below the limit of quantification, by a maximum-likelihood method involving left-censoring at the LOD/LOQ. The red vertical line shows the statutory maximum residue level (MRL) for poultry and other non-game meat.

**Figure 3:**
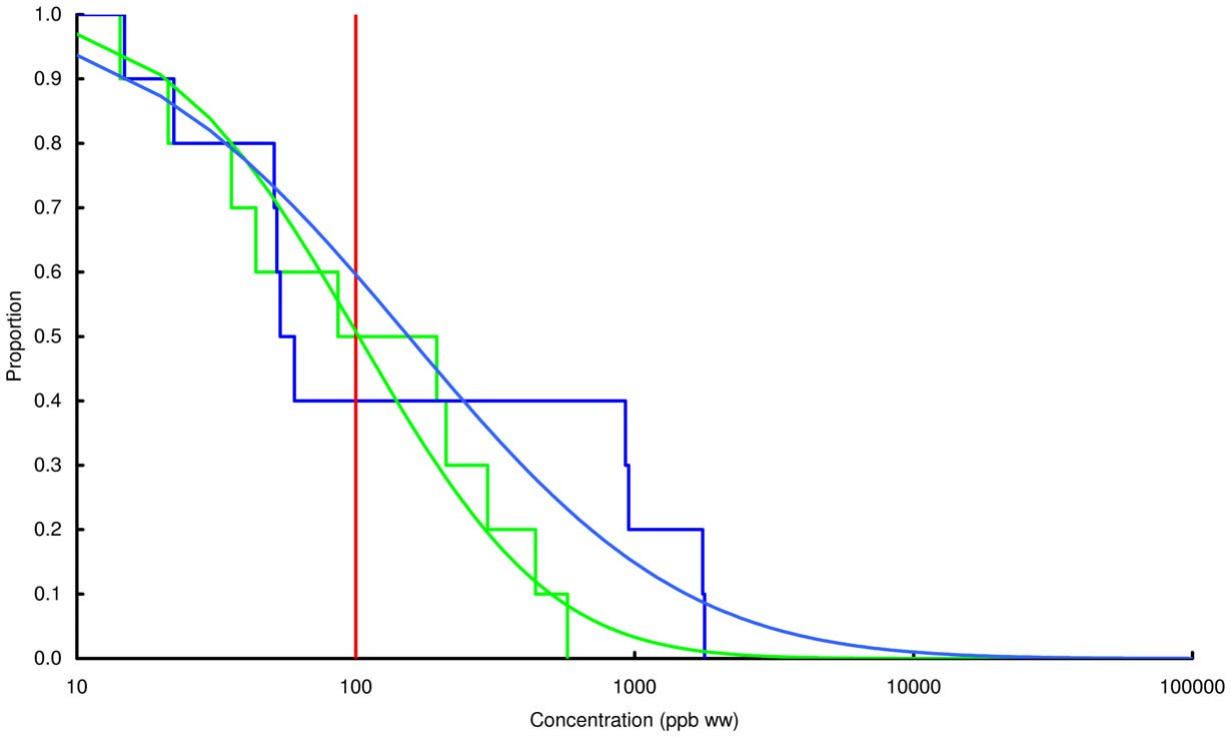
Exceedance distribution of lead concentrations in red grouse. Observed (stepped line) and fitted (curve) exceedance distribution of lead concentrations (ppb wet weight) in red grouse cooked using acidic (green) and non-acidic (blue) methods. The curves represent lognormal distributions fitted to all the data, including those below the limit of quantification, by a maximum-likelihood method involving left-censoring at the LOD/LOQ. The red vertical line shows the statutory maximum residue level (MRL) for poultry and other non-game meat.

**Figure 4:**
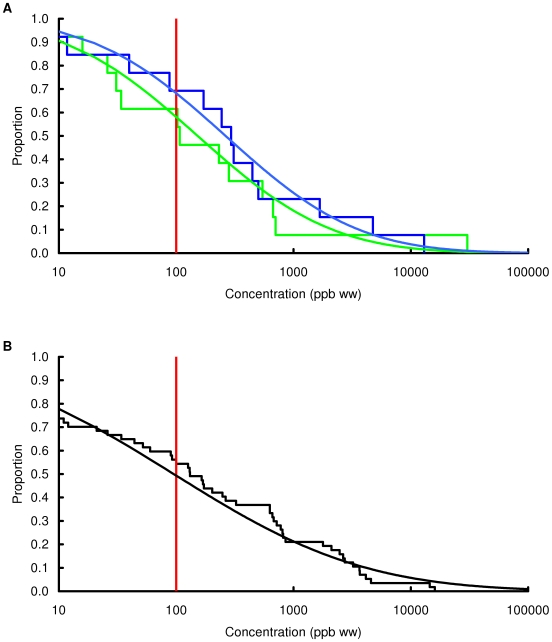
Exceedance distribution of lead concentrations in partridge from the WWT and VMD studies. Observed (stepped line) and fitted (curve) exceedance distribution of lead concentrations (ppb wet weight) in partridge (A) cooked using acidic (green) and non-acidic (blue) methods and (B) fresh uncooked tissue from the VMD statutory surveillance programme. The curves represent lognormal distributions fitted to all the data, including those below the limit of quantification, by a maximum-likelihood method involving left-censoring at the LOD/LOQ. The red vertical line shows the statutory maximum residue level (MRL) for poultry and other non-game meat.

**Figure 5:**
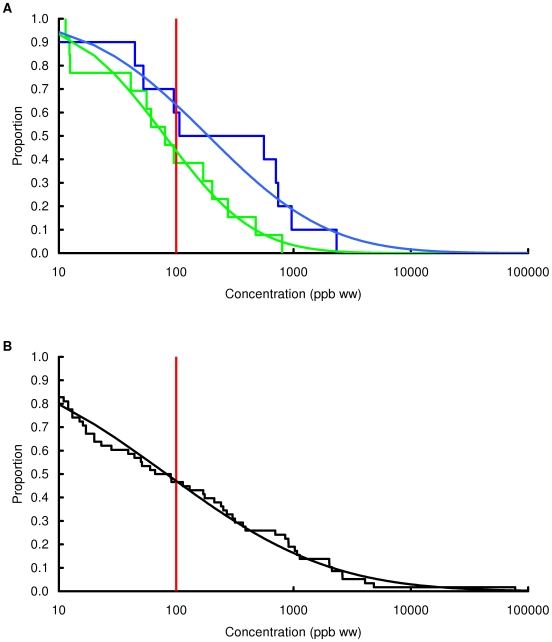
Exceedance distribution of lead concentrations in pheasant from the WWT and VMD studies. Observed (stepped line) and fitted (curve) exceedance distribution of lead concentrations (ppb wet weight) in pheasant (A) cooked using acidic (green) and non-acidic (blue) methods and (B) fresh uncooked tissue from the VMD statutory surveillance programme. The curves represent lognormal distributions fitted to all the data, including those below the limit of quantification, by a maximum-likelihood method involving left-censoring at the LOQ. The red vertical line shows the statutory maximum residue level (MRL) for poultry and other non-game meat.

**Figure 6:**
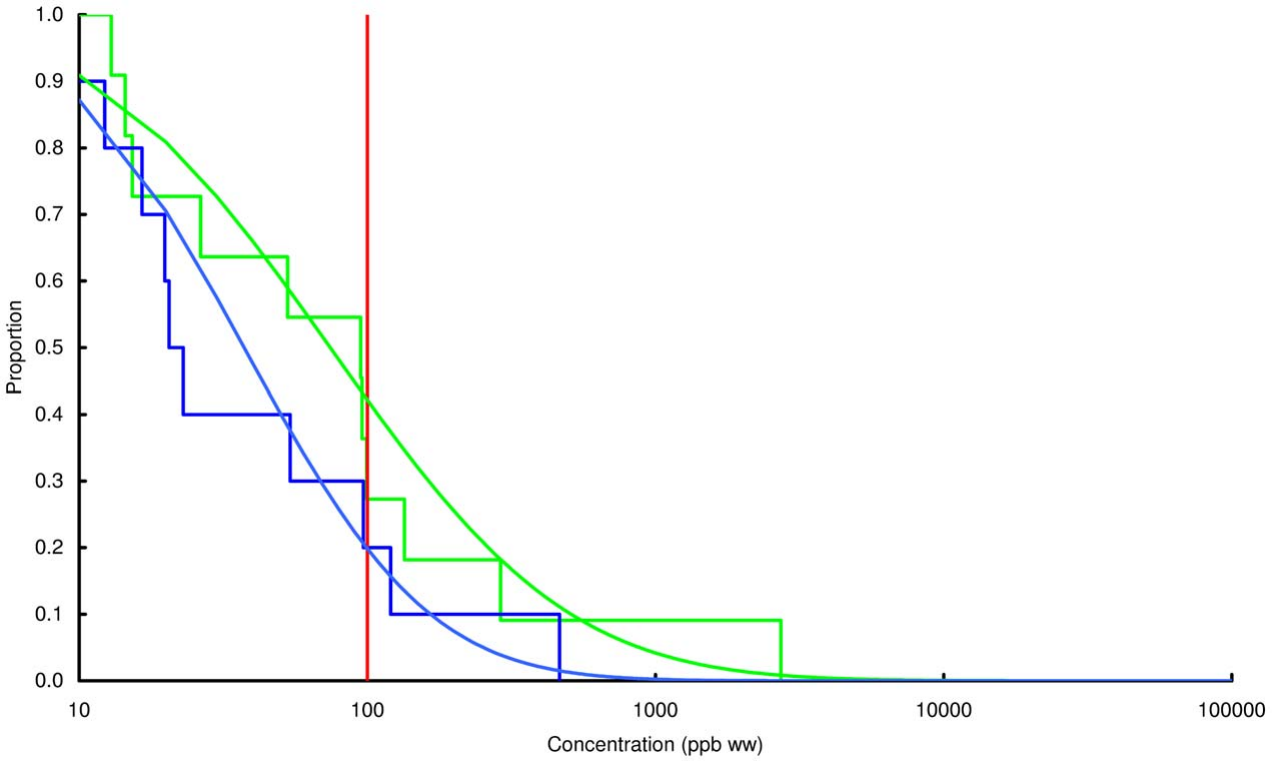
Exceedance distribution of lead concentrations in woodpigeon. Observed (stepped line) and fitted (curve) exceedance distribution of lead concentrations (ppb wet weight) in woodpigeon cooked using acidic (green) and non-acidic (blue) methods. The curves represent lognormal distributions fitted to all the data, including those below the limit of quantification, by a maximum-likelihood method involving left-censoring at the LOD/LOQ. The red vertical line shows the statutory maximum residue level (MRL) for poultry and other non-game meat.

**Figure 7:**
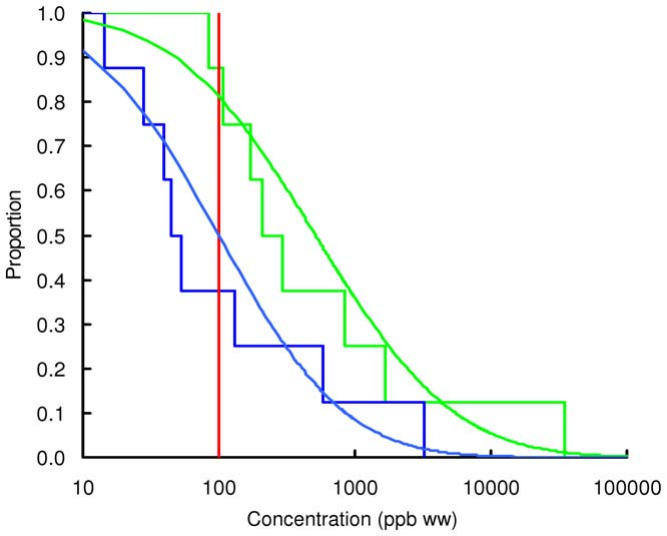
Exceedance distribution of lead concentrations in woodcock. Observed (stepped line) and fitted (curve) exceedance distribution of lead concentrations (ppb wet weight) in woodcock cooked using acidic (green) and non-acidic (blue) methods. The curves represent lognormal distributions fitted to all the data, including those below the limit of quantification, by a maximum-likelihood method involving left-censoring at the LOD/LOQ. The red vertical line shows the statutory maximum residue level (MRL) for poultry and other non-game meat.

**Figure 8:**
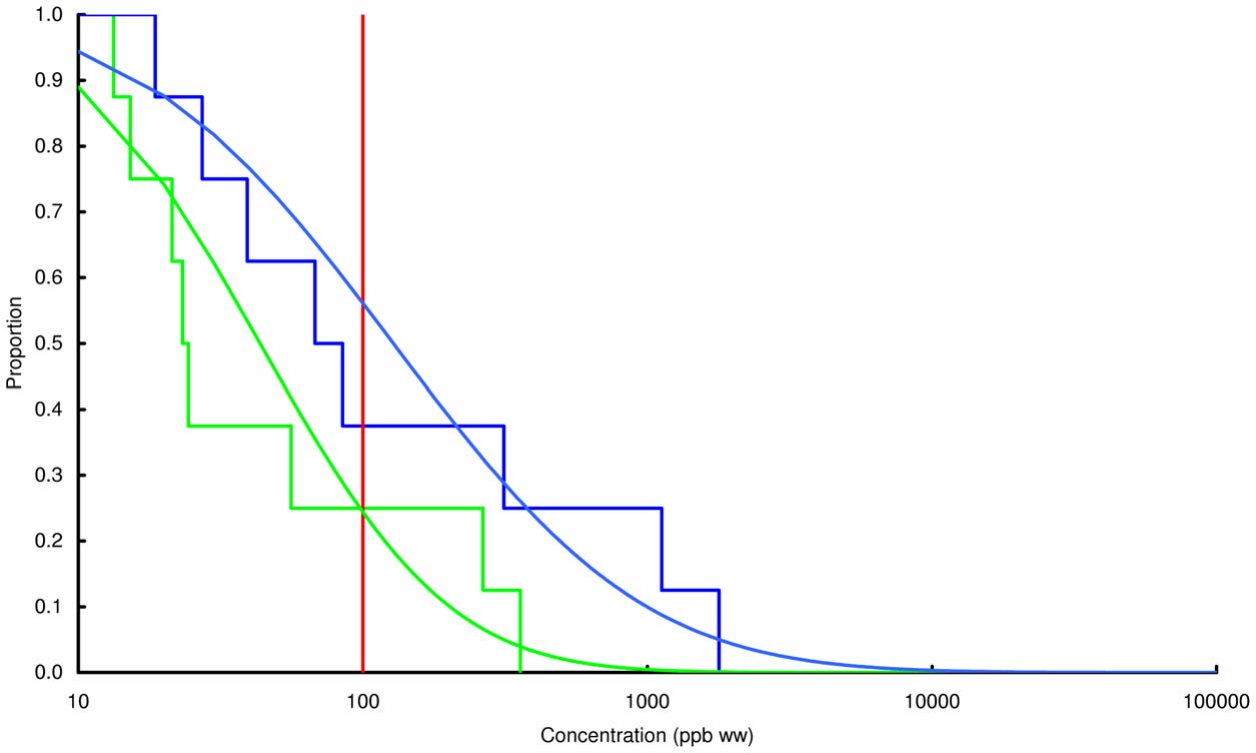
Exceedance distribution of lead concentrations in wild mallard. Observed (stepped line) and fitted (curve) exceedance distribution of lead concentrations (ppb wet weight) in wild mallard cooked using acidic (green) and non-acidic (blue) methods. The curves represent lognormal distributions fitted to all the data, including those below the limit of quantification, by a maximum-likelihood method involving left-censoring at the LOD/LOQ. The red vertical line shows the statutory maximum residue level (MRL) for poultry and other non-game meat.

**Figure 9:**
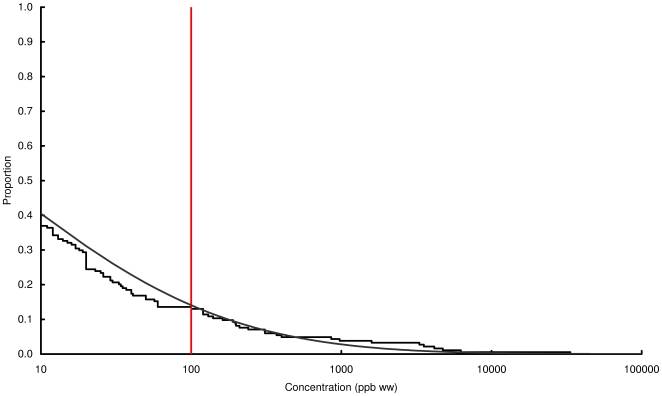
Exceedance distribution of lead concentrations in wild deer from the VMD statutory surveillance programme. Observed (stepped line) and fitted (curve) exceedance distribution of lead concentrations (ppb wet weight) in fresh uncooked venison tissue from wild deer from the VMD statutory surveillance programme. The curves represent lognormal distributions fitted to all the data, including those below the limit of quantification, by a maximum-likelihood method involving left-censoring at the LOD/LOQ. The red vertical line shows the statutory maximum residue level (MRL) for poultry and other non-game meat.

Observed and modelled percentages of samples exceeding three thresholds, 100, 1000 and 10000 ppb by wet weight, are shown in Table 3 and the modelled and observed distributions of values are in Figures 2, 3, 4, 5, 6, 7, 8, 9. Very small proportions of chicken samples exceeded the 100 ppb wet weight threshold and none exceed the other two thresholds. However, substantial proportions of samples from gamebirds and deer exceeded the 100 ppb threshold and some samples of all game species exceeded the 1000 ppb threshold. Some measurements from partridge, pheasant, woodcock and deer exceeded the 10000 ppb threshold. Results from the WWT study and VMD statutory surveillance programme for the two species covered by both studies (partridge and pheasant) were broadly similar (Table 3, Figures 4 and 5).

**Table 3:** Percentages of samples of game and chicken that exceeded each of three threshold values of lead concentration (100, 1000 and 10000 ppb wet weight).

		Percent exceeding		Percent exceeding		Percent exceeding		
		100 ppb ww		1000 ppb ww		10000 ppb ww		
Species	Cooking method	Observed	Modelled	Observed	Modelled	Observed	Modelled	*N*
Chicken	Acid	0	0.1	0	0	0	0	14
Chicken	Non-acid	2.4	0.6	0	0	0	0	42
Red grouse	Acid	50	50.8	0	3.3	0	0	10
Red grouse	Non-acid	40	59.6	20	14.9	0	1	10
Partridge	Acid	61.5	58.1	7.7	18.1	7.7	2.1	13
Partridge	Non-acid	69.2	68.2	23.1	25.7	7.7	3.8	13
Partridge*	Fresh	56.1	47	21.3	20.3	4.6	5.7	57(108)
Pheasant	Acid	38.5	43.7	0	3.4	0	0	13
Pheasant	Non-acid	60	63.3	10	18.3	0	1.6	10
Pheasant*	Fresh	46.6	43.4	17.9	14.2	0.9	2.4	58(106)
Woodpigeon	Acid	27.2	42.2	9.1	4.2	0	0.1	11
Woodpigeon	Non-acid	20	19.9	0	0.2	0	0	10
Woodcock	Acid	87.5	81.2	25	35.9	12.5	5.4	8
Woodcock	Non-acid	37.5	49.8	12.5	8.5	0	0.3	8
Mallard	Acid	25	24.4	0	0.4	0	0	8
Mallard	Non-acid	37.5	56.1	25	10	0	0.3	8
Deer*	Fresh	13.6	15.8	4.4	3.7	0.4	0.5	184(271)

*VMD data. All other data are from the WWT study.

Results were derived from two independent studies. In one study by WWT, chickens and gamebirds were prepared and cooked using methods characteristic of the UK involving or not involving acidic marinades or sauces. Concentrations of lead were derived from analyses of meat and sauce separately, which were combined to give an estimate of wet weight concentration in the meat and sauce combined. The other study was the statutory surveillance programme of the Veterinary Medicines Directorate (VMD) and involves analyses of aliquots from homogenised fresh carcasses. In each case, the observed percentage of samples with concentrations exceeding the threshold is given, together with an estimate from a fitted lognormal distribution with left-censoring at the limit of quantification (LOD, or LOQ for VMD data). Values for the VMD samples with concentrations below 300 ppb were not reported for the period 2001–2003, so the observed proportion exceeding 100 ppb is given for the period 2004–2008. Sample sizes are shown for 2004–2008, and in brackets for 2001–2008.

Analysis of the WWT data indicated no significant effect of cooking method in any of the bird species (likelihood-ratio tests, *P*>0.20 in all species). Inspection of Figures 2, 3, 4, 5, 6, 7, 8 reveals that the non-significant differences were in different directions for different species. There was a highly significant difference in the distributions of lead concentrations among the seven bird species (likelihood-ratio test, χ^2^_(12)_ = 132.66, *P*<0.0001), but this difference was due entirely to inclusion of chicken in the group of species sampled. When chicken was excluded, there was no significant variation across the six gamebird species (likelihood-ratio test, χ^2^_(10)_ = 3.29, *P* = 0.974).

Modelled percentages of samples exceeding three thresholds, 100, 1000 and 10000 ppb by wet weight, are shown in Table 4 for birds pooled across cooking methods. Also shown are the adjusted equivalent estimates for the proportions of exceedances expected had the lead concentration been determined from all the meat from each bird, rather than a small aliquot. It can be seen that this adjustment for the effect of within-bird, among-aliquot variation reduces the proportion of exceedances in most cases, especially for the high thresholds. However, the effect is quite small and the overall conclusion that a substantial proportion of samples exceed the lower two thresholds is still supported by this analysis. In the VMD statutory surveillance programme there was averaging across multiple aliquots for samples whose initial screening sample gave a high concentration, but not for screening samples with low concentrations. This procedure makes it impractical to make an adjustment for within-animal, among-aliquot variation in the VMD data. However, taking multiple aliquots for animals with high screening values will tend to reduce the proportion of exceedances, as does our adjustment of the WWT data.

**Table 4:** Percentages of samples of cooked gamebirds and chicken estimated from fitted lognormal models to have exceeded each of three threshold values of lead concentration (100, 1000 and 10000 ppb wet weight).

	Percent exceeding		Percent exceeding		Percent exceeding	
	100 ppb ww		1000 ppb ww		10000 ppb ww	
Species	Not adjusted	Adjusted	Not adjusted	Adjusted	Not adjusted	Adjusted
Chicken	0.5	0.1	0.0	0.0	0.0	0.0
Red grouse	55.9	54.6	9.1	6.4	0.2	0.1
Partridge	63.1	63.4	21.9	19.8	3.0	2.0
Pheasant	54.0	53.1	10.0	8.0	0.4	0.2
Woodpigeon	32.7	29.1	1.8	0.8	0.0	0.0
Woodcock	66.2	66.8	22.2	19.5	2.6	1.6
Mallard	42.3	39.9	4.3	2.4	0.1	0.0

All samples are from the WWT study. Estimates with and without an adjustment to allow for within-bird variation in concentration among aliquots of meat are shown. The adjusted values approximate what would have been expected if the measurements of concentration in the whole meal derived from each bird had been available. The raw values are equivalent to the modelled values in Table 3, but data for acid and non-acid cooking methods have been pooled for each species.

### Potential dietary exposure to lead from meals made from wild game

We simulated the exposure to lead from dietary sources of a person in the UK of 70 kg bodyweight using estimates of typical quantities of lead ingested with the diet [28, Table 4.2]. According to this source, a typical UK adult has a weekly lead intake of 0.186 mg of which 0.0086 mg is derived from the average consumption of 88 g (ww) of meat per day [28, Table 4.1]. The lead intake derived from dietary sources other than meat is therefore 0.177 mg per week. The total weekly dietary intake of lead represents about 11% of the FAO/WHO Provisional Weekly Tolerable Intake (PTWI) for a 70 kg adult, which is 1.75 mg (0.025 mg kg^−1^ body weight) [29]. We simulated the effect on dietary lead intake of replacement of the meat in the typical UK diet by meals based upon the meat of chicken and game animals covered by the VMD statutory surveillance programme and WWT surveys of lead concentration. We did this in two ways.

1. We assumed that the person ate meals each of which was based upon 150 g (ww) of meat. The size of the portion of meat we used was based upon the estimate of 5 ounces (141 g) by Kosnett [30] and the estimate by Haldimann and colleagues [31] who considered that 2.2 meals based upon game meat per week was equivalent to 50 g of game meat per day (i.e. 159 g per meal). We calculated the lead content of such a meal from the arithmetic mean lead concentration, per unit weight of meat, for the species concerned, including the lead associated with both the meat and sauce components of the meal, for those meals that included sauce. We then calculated how many such meals would need to be eaten for the lead intake from the combined meals to equal the difference between the PTWI for a 70 kg adult and the weekly lead intake from dietary sources other than meat. This is the number of meat meals required to cause the person to exceed the PTWI.
2. We assumed that the typical intake of meat by a UK adult (88 g d^−1^) was replaced by meals based upon 88 g of meat per day of the species concerned. We calculated the lead content of these meals as described above and then expressed the simulated total weekly intake of lead from all dietary sources as a percentage of the PTWI.

These levels of game consumption should be regarded as a worst case. However, they are feasible for people who shoot game animals or have access to them at low or no cost because of their employment on game-shooting estates or elsewhere in the industry and are able to store a seasonal surplus of game meat by freezing it. The results indicate that adults in the UK who consume unusually high quantities of game meat from some species might exceed the FAO/WHO PTWI (Table 5). Species for which a daily meal based upon 150 g of meat would cause the PTWI to be exceeded are partridge (VMD data), woodcock (WWT) and pheasant (VMD). The alternative method of calculation based on the average meat component of the adult UK diet being replaced by game led to the PTWI being exceeded for consumption of partridge (VMD data) and woodcock (WWT). Red grouse consumption at these levels would cause the PTWI to be approached, though not exceeded. Consumption of mallard, woodpigeon and deer appear to pose a smaller risk of PTWI exceedance. The sizes of the WWT samples are small. Estimates of the arithmetic mean lead concentration are not particularly precise unless the sample size is large. However, it should therefore be noted that the large samples of partridges and pheasants derived from VMD statutory surveillance programme are among those giving the greatest cause for concern.

**Table 5:** Effect on the weekly dietary lead exposure of a 70 kg person of replacing the typical meat component of the diet in the UK with meals of meat of each of several species, including game animals.

Source	Species	*N*	Arithmetic mean concentration in whole meal per unit of meat (ppb)	Number of 150 g meals per week to exceed PTWI	Percentage of PTWI from whole diet if 88 g of meat eaten per day
WWT	Chicken	56	19.1	546.8	11.0
WWT	Red grouse	20	1165.2	9.0	51.3
WWT	Mallard	16	341.3	30.7	22.3
WWT	Partridge	26	1120.1	9.3	49.7
VMD	Partridge	108	8054.1	1.3	293.8
WWT	Pheasant	23	979.6	10.7	44.8
VMD	Pheasant	106	1613.6	6.5	67.1
WWT	Woodpigeon	21	433.0	24.2	25.5
WWT	Woodcock	16	3410.5	3.1	130.4
VMD	Deer	271	377.4	27.7	23.6

The estimated arithmetic mean concentration of lead in foods is expressed in ppb by wet weight of meat, with the lead present in the sauce of WWT samples cooked with sauce also being included. The number of meals of each species, based upon 150 g of meat, that would need to be consumed for the weekly intake of lead from the whole diet to exceed the FAO/WHO Provisional Weekly Tolerable Intake (PTWI) is given. Also given is the weekly lead intake from the whole diet, as a percentage of PTWI, if the typical daily consumption of meat in the UK (88 g d^−1^ ww) was replaced by a daily meal based upon 88 g of meat of the species shown.

It should also be borne in mind that the FAO/WHO PTWI may be set somewhat higher than some experts on the human health effects of lead would now consider appropriate and that groups who eat unusually high amounts of game may also eat game with higher lead concentrations than indicated by these results if they eat animals of low market value because of damage by shot.

### Concentration of lead in gamebird tissue in relation to the number and composition of gunshot pellets and shot fragments in the carcass

**Figure 10:**
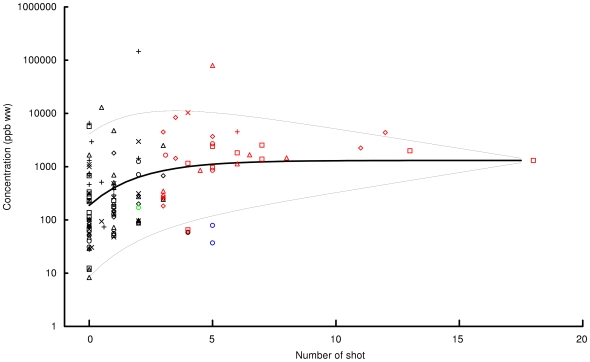
Lead concentration in gamebirds in relation to the number of gunshot pellets in the carcass. Concentration of lead (ppb) per unit mass of cooked meat (wet weight) of gamebirds in relation to the number of gunshot pellets detected on X-rays of the carcass. Each symbol represents results for one bird with species denoted as follows: diamond = red grouse, circle = mallard, triangle = partridge, square = pheasant, x = woodpigeon, + = woodcock. Black symbols denote samples for which the composition of the shot was not assayed or for which colorimetric analysis was inconclusive (1 bird). Red symbols denote samples in which the colorimetric analysis detected lead. Blue symbols denote samples in which lead was not detected and in which the shot were considered to be composed primarily of bismuth alloy and the green symbol a bird from which only steel shot were recovered. The thick curve shows the fitted relationship between concentration and pellet number and the thin curves show the 5^th^ (lower) and 95^th^ percentiles (upper) of the distribution, estimated from the model fitted to the data, excluding the three birds considered to have been shot only with bismuth alloy or steel.

We investigated the relationship between the number of gunshot detected by X-ray in the carcass of the gamebirds from the WWT sample and the concentration of lead in the whole meal (meat and sauce) prepared from it, expressed as concentration per unit mass of cooked meat. Gamebirds with more than five shot detected in the carcass had high concentrations of lead in the cooked meal (> about 1000 ppb), except for two mallard which had been shot with ammunition which contained no detectable lead, and were suspected to be bismuth alloy (Figure 10). Shot containing lead were present in all but one of the other carcasses for which the presence or absence of lead in pellets was determined.

There was a highly significant positive relationship between lead concentration in the cooked meal and the number of gunshot pellets in the carcass, excluding two birds that contained bismuth alloy shot and one with only steel shot present (Spearman rank correlation coefficient *r_S_* = 0.484, *P*<0.0001). However, the relationship was not a simple one of progressive increase of lead concentration with increasing pellet numbers. Lead concentration tended to increase with pellet numbers most rapidly at low pellet numbers and then level off (Figure 10). There was also a marked tendency for lead concentration to become less variable as pellet numbers increased. The maximum-likelihood analysis gave the asymptotic relationship between lead concentration and pellet number as 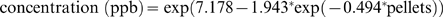, with the residual standard deviation of log concentration about the mean being given by 1.878–0.104*pellets. Deletion from this model of the effect of pellet number on geometric mean concentration indicated a highly significant relationship (likelihood-ratio test; *χ^2^* = 45.73, d.f. = 2, *P*<0.0001). The negative relationship between residual standard deviation and pellet number was also significant (likelihood-ratio test; *χ^2^* = 13.79, d.f. = 1, *P* = 0.0002). The fitted model indicates a large decrease in variability of lead concentration as pellet numbers increased. The 95^th^ percentile lead concentration was 482 times the 5^th^ percentile in birds with no visible pellets, but this ratio was only three in birds with 15 pellets.

This analysis was developed further by including the effect upon lead concentration of the ordinal scale representing the number of small radio-dense fragments visible on the X-rays. As in the analysis reported above, data from the three mallard known to have been killed using non-lead shot were excluded. Lead concentration in cooked meat was significantly positively correlated with the small fragment score on its own (*r_S_* = 0.409, *P*<0.0001). However, the small fragment score was positively correlated with the number of shot and large fragments visible on X-ray (*r_S_* = 0.351, *P* = 0.0001). Hence, the correlation of lead concentration with small fragment score might be spurious and really due to the effect of the number of shot and large fragments already described. To assess the influence of small fragments, large fragments and shot together, we adapted the maximum-likelihood model described above as described in the Methods. The analysis revealed a significant positive effect of small fragment score on lead concentration when both it and the number of gunshot pellets were included in the model. The fitted model was 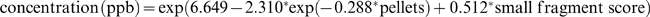. For this formulation, there was no significant tendency for the residual variation in log lead concentration to decline with increasing pellet numbers (likelihood-ratio test; *χ^2^* = 0.83, d.f. = 1, *P* = 0.363), so a constant residual standard deviation was estimated as 1.473. Deletion from the model of each of the effects of the number of pellets visible on X-ray (likelihood-ratio test; *χ^2^* = 22.59, d.f. = 2, *P*<0.0001), and the small fragment score (*χ^2^* = 9.91, d.f. = 1, *P* = 0.0016), resulted in highly significant increases in residual deviance. There was a reasonably high correlation between observed and modeled log concentrations when both the number of shotgun pellets and the small fragment score were included in the model (Pearson correlation coefficient, *r* = 0.550) The correlation between observed and expected values was weaker when only the number of shotgun pellets (*r* = 0.492) or the small fragment score (*r* = 0.395) were used on their own as independent variables. Hence, both the number of shotgun pellets and large fragments, which were removed after cooking, and the number of small fragments, which were not, had significant separate effects on lead concentration in the cooked meal.

### Proportion of lead required to be lost as fragments from gunshot entering the body of a gamebird to account for the concentration of lead in cooked gamebird tissue

We made an approximate calculation of the proportion of the mass of lead in the gunshot that strike a gamebird that would need to fragment into small particles to account for the concentration of lead per unit mass of cooked meat in our analysis of meals derived from gamebirds. To do this, we assumed that all the birds were killed with number 6 lead shot, and that all of the lead in birds was from gunshot fragments. We multiplied the mean number of shot per bird from X-rays by the mass of a number 6 shot (0.126 g) and divided that by the mean body mass of each gamebird species to obtain the concentration of lead shot in the body per unit mass of all tissues. We then took the ratio of the concentration of lead per unit mass of cooked meat from our lead assays to the concentration of lead shot in the body in gunshot per unit body mass to be the proportion of lead lost from gunshot pellets as small fragments. Species-specific estimates of this proportion ranged from 0.163% to 0.812% and averaged 0.308%. (Table 6).

**Table 6:** The mass of lead in small fragments within the body of a gamebird as a percentage of the mass of gunshot that entered the body.

	Red grouse	Mallard	Partridge	Pheasant	Woodpigeon	Woodcock
**Shot pellets per bird**	3.050	2.313	2.115	3.318	0.952	1.000
**Mass of lead shot per bird (g)**	0.384	0.291	0.267	0.418	0.120	0.126
**Body mass (g)**	600	1063	488	1163	453	300
**Lead shot per unit body mass (ppb ww)**	640490	274231	546737	359642	265189	419993
**Lead in cooked meat (ppb ww)**	1165	593	1120	980	433	3410
**Lead in meat as percentage of that in shot**	0.182	0.216	0.205	0.272	0.163	0.812

The calculations assume that all birds were shot with number 6 lead shot. The mean concentration in cooked meat from mallards excludes two birds killed using bismuth alloy shot and one with only steel shot present.

## Discussion

Our analyses of meals prepared using meat from wild-shot gamebirds showed that the concentration of lead in meals prepared from the flesh was significantly correlated with the number of shot visible on X-rays, even though apparently intact shot and large fragments were removed after cooking. All birds with many lead shot in the carcass had high levels of lead in the meal prepared from them, but some birds with few or no shot visible on X-rays also had high lead concentrations in the meal. This can be explained by the presence of the variable numbers of tiny radio-dense fragments, presumed to be lead, which we observed on X-rays of the carcasses of a high proportion of birds, including the majority of those in which all gunshot had passed through the body. Our X-rays were not of very high resolution, so we suspect that we failed to detect small metallic fragments in many of the birds which appeared to have none. We suggest that the high variance in lead concentration for birds with low numbers of shot in the carcass may have resulted from the variable fragmentation of shot, perhaps associated with variation in projectile velocity or whether or not the shotgun pellet struck bone.

It could be argued that some of the lead we measured in the meals could have originated from sources other than lead from projectiles shot into the body, such as lead absorbed from ingested lead shot, or from exposure to locally elevated sources of lead [e.g. 32]. However, these contributions are likely to be very small as the highest concentrations of biologically incorporated lead in birds are generally found in bone, kidney and liver tissue, whereas the lowest concentrations are found in muscle and fat [e.g. 33 and references therein], which were the tissues used to prepare meals in our study. Even in birds that have been experimentally exposed to high levels of ingested lead gunshot, muscle levels of lead were relatively low (ca. 200–300 ppb w.w. in bald eagles *Haliaeetus leucocephalus* that died after dosing with ten lead shot and ca.<20–35 ppb ww in birds that survived but were euthanised [34]). Furthermore, if anything other than a minor part of the lead we measured in gamebird meals had come from ingested gunshot or other environmental contamination, then the highly significant correlation we observed between lead concentration in the meal and the number of shot and small fragments visible on X-rays (Fig 10) would have been obscured. Hence, the most convincing explanation for these results is that the lead measured in the meals was derived from lead gunshot pellets shot into or through the birds' bodies. The low tissue lead concentration found in two mallards killed using bismuth alloy shot is also consistent with this explanation.

Although the number of gunshot per bird observed on X-rays varied significantly among gamebird species, the mean concentration of lead did not. This may be because the mean number of gunshot per bird was positively related to the mean body mass of the species. Hence, large gamebird species contained more gunshot per individual, but the number of shotgun pellets per unit mass of tissue, and the lead concentration derived from them, might vary less. Consistent with this suggestion, we found a high, though non-significant, correlation across gamebird species between the arithmetic mean concentration of lead per unit mass of cooked meat and the mean number of shot per unit body mass (Spearman rank correlation coefficient *r_S_* = 0.771).

Our results indicate that, as has been shown previously in ducks and North American grouse [35], [36], lead gunshot undergoes sufficient fragmentation on impact with gamebirds and that lead fragments cause contamination of their meat and, hence, increased exposure to lead of human consumers of game. An approximate calculation suggests that about 0.3% of the mass of lead shot that strike a gamebird fragment into small particles that are unlikely to be removed during food preparation or by the consumer. The calculation is approximate because the concentration of lead in cooked meat is taken to be equivalent to that in fresh tissue and because it is assumed that the number of shot entering the body is the same as the number of shot and large fragments found on X-rays. In fact, it is likely that some shot exit the body leaving small fragments, so the percentage loss estimate is likely to be a maximum. Hence, it seems that only a small proportion of the lead in gunshot striking gamebirds is required to fragment into small particles of lead in order to account for the lead concentrations found in cooked meat in our study. Experiments could be performed under controlled conditions to determine the fate of lead from gunshot pellets that strike gamebirds and thereby refine these rough estimates.

A high proportion of tissue of both cooked and raw gamebirds and raw venison (all excluding offal) had lead concentrations that exceed the EU ML of 100 ppb wet weight for lead in bovine animals, sheep, pigs and poultry. In several game species, a high proportion of samples exceeded ten times the ML, with a few samples exceeding 100 times the ML. Concentrations of lead in the meat of partridges and pheasants were broadly similar and not significantly different between the VMD and WWT samples. At first sight, this is surprising because whole gunshot and large fragments were dissected out before homogenization and analysis for the WWT samples, but not for the VMD samples. For the assessment of dietary exposure, we consider it preferable to remove gunshot and large fragments before analysis because that simulates what consumers are likely to do. However, it should be noted that a duplicate aliquot was analysed for all VMD samples where the lead concentration of the first aliquot was high and a mean value taken, but a second aliquot was not analysed for VMD samples with low lead levels in the first aliquot. This procedure, which was not performed for WWT samples, would tend to reduce the tendency for the presence of gunshot and large fragments in the VMD samples to increase measured lead concentrations above those in the WWT samples. Lead concentrations found in pheasants in our study were broadly comparable with those found in a previous survey commissioned by the UK Food Standards Agency [37], [38]. In that study, only 12 pheasants were sampled, but the percentages of birds with lead concentrations exceeding the 100, 1000 and 10000 ppb (ww) thresholds were 83.3%, 8.3% and 0% respectively. This compares with 47.8%, 4.4%, 0% observed threshold exceedances for the WWT data and 46.6%, 17.9% and 0.9% for the VMD data.

Lead concentrations in cooked meals were not significantly affected by the cooking method used, when typical UK cooking methods were employed. In particular, whether or not recipes included acidic ingredients (wine or cider) did not affect mean lead concentration. Lead is more soluble under acidic conditions and it has been shown that, when lead shot are present, the southern European practice of cooking game meat in vinegar increased the concentration of lead in the edible tissues of game with manually embedded lead shot [39]. However, it may be that the acidity of the sauces used in UK recipes or the duration of cooking of meat in them were lower than in Spain.

The effect on the weekly dietary intake of lead by adults in the UK of consuming meals based upon game meat with the concentrations of lead found in this paper were simulated by modifying the typical UK diet. It was found that unusually high but feasible levels of consumption of partridge, woodcock and pheasant meat could result in the current FAO/WHO Provisional Weekly Tolerable Intake (PTWI) of lead for a 70 kg adult being exceeded. High levels of consumption of red grouse meat might also cause the PTWI to be approached. The level of absorption of dietary lead assumed in setting the PTWI may differ from that which applies for particles of metallic lead derived from lead ammunition. Absorption by rats of lead from large particles (180–250 µm diameter) of metallic lead is lower than from lead salts and biologically incorporated lead [22]. However, smaller particles of metallic lead are more readily absorbed, especially those <50 µm diameter [40]. In addition, cooking may change the chemical form of some of the metallic lead, also influencing availability. Hence, the size distribution of particles of metallic lead in game animals and the degree to which lead from fragments of ammunition is dissolved or otherwise altered by cooking techniques should be taken into account in assessing the risk posed by lead ammunition.

In comparing potential dietary intake rates with current PTWI values it is important to recognise that recent research findings of adverse health effects at very low blood lead concentrations have led health experts to call for a halving in the current blood lead action threshold [10], [11]. For example, Chandramouli and colleagues [10] found that in children 30 months old blood lead levels of 5–10 µg dL^−1^ were associated with a reduction in SAT (Standard Assessment Test) grades, supporting the results of other recent studies that have reported effects on behaviour and cognition in children of blood lead levels <10 µg dL^−1^ [8], [9], [41], [42]. These results also have implications for the FAO/WHO PTWI and for MLs of lead for foodstuffs set within Regulation EC 1881/2006. The current PTWI for lead of 0.025 mg kg^−1^ body weight was set in 1982 for infants and children, extended to all age groups in 1993, and has not changed since [29], whilst blood lead action thresholds have progressively declined. MLs of contaminants in foods are reviewed regularly, and, at the request of the European Commission, the European Food Safety Authority (EFSA) Scientific Panel on Contaminants in the Food Chain is currently developing a scientific opinion on the risks to human health related to the presence of lead in foodstuffs. This will consider new information on the toxicity of lead and updated the assessment of exposure from food. While it may be considered impractical to set MLs for contaminants in all foodstuffs, we believe that there is a compelling case for including wild game meat within Commission Regulation EC 1881/2006 [12]. Wild-shot game is widely consumed across the UK, is actively marketed and sold in high-volume retail outlets and shows an increasing trend in consumption [23]. A previous UK risk assessment [26], which concluded that the risk from dietary lead derived from game meat was low, was based upon average game consumption levels derived from a small survey and ignored the highly skewed distribution of dietary intake rates. Certain groups of consumers, such as those who prefer wild food for its presumed health and welfare advantages may have levels of consumption of game considerably higher than the average. People involved in the management of game animals may be particularly at risk from lead contamination of game meat. An unpublished survey commissioned by the ‘Game to Eat’ campaign estimated that 14% of the game shot in Britain is given away or sold informally, mostly to shooters, beaters and other shoot helpers (http://www.epolitix.com/Resources/epolitix/Forum%20Microsites/Countryside%20Alliance/CA_280704.pdf). Assuming that most of this meat is consumed by this relatively small group and their families, this implies a high level of per capita consumption. In addition, it seems likely that game disposed of in this way may be of lower than average market value due to visible damage caused by impacts from large numbers of gunshot. If these animals contain more lead shot than those sent to market then, as suggested by our results, average lead concentrations in game consumed by this putative high-exposure group may also be higher than the average indicated by our analyses of meat from marketed game. A thorough and quantitative analysis of the potential risks to human health from the consumption of game shot with lead ammunition is urgently required. Such risk analysis should include an evaluation not only of groups with higher than average exposure, but also those with high vulnerability to the effects of a given amount of ingested lead (i.e. young children and pregnant women).

## Supporting Information

Text S1 Recipes used to cook gamebirds and chicken controls.
(0.06 MB DOC)
