# Supplementary material for: Potential Hazard to Human Health from Exposure to Fragments of Lead Bullets and Shot in the Tissues of Game Animals
Source: PLoS One. 2010 Apr 26;5(4):e10315. doi: 10.1371/journal.pone.0010315 (PMC2859935; doi:10.1371/journal.pone.0010315)
Supplement: Text S1 — Recipes used to cook gamebirds and chicken controls. (0.06 MB DOC) [file pone.0010315.s001.doc]

Roast Pheasant[1](http://mail.wwt.org.uk/owa/WebReadyViewBody.aspx?t=att&id=RgAAAABoQbhgsAJASrxAAS%2BIFHZBBwAKwNyneWHaQpCHYc28VTSqAAMRDJ63AAAKwNyneWHaQpCHYc28VTSqAVHwWVfGAAAJ&attid0=EABNIObF8FJORay2d0Bj8Kn2&attcnt=1&pn=1#footnote1%23footnote1)

#### Ingredients

#### **1 pheasant**

#### **1 onion**

#### **Bacon fat**

# Method

1. Draw and truss the bird.

2. Insert half an onion in the bird, also a piece of bacon fat.

3. Wrap the bird in bacon fat and wrap loosely in foil.

4. Place in hot oven for 30 minutes, reduce to 176ºC and cook for 40 minutes.

Pheasant casserole with cider[2](http://mail.wwt.org.uk/owa/WebReadyViewBody.aspx?t=att&id=RgAAAABoQbhgsAJASrxAAS%2BIFHZBBwAKwNyneWHaQpCHYc28VTSqAAMRDJ63AAAKwNyneWHaQpCHYc28VTSqAVHwWVfGAAAJ&attid0=EABNIObF8FJORay2d0Bj8Kn2&attcnt=1&pn=1#footnote2%23footnote2)

**Ingredients**

1 pheasant

121 g onions, sliced

1 large clove garlic, finely chopped

159 g carrots, diced

120 g celery, sliced

83 g mushrooms, sliced

568 ml cider

1 tbsp olive oil

1 bay leaf

1 bouquet garni

Seasoning (2 shakes of salt and 2 shakes of pepper)

**Method**

1. Pre-heat oven to 150ºC. Heat oil in a large casserole. Brown the meat evenly.

2. Soften onion, celery and garlic for five minutes in the casserole.

3. Add the mushrooms to the pot, along with the browned meat.

4. Season.

5. Add the cider so that the ingredients are covered.

6. Add bay leaf and bouquet garni.

7. Turn heat up a little and bring to simmering point - give a good stir.

8. Place in the oven and cook at 150ºC for 60 minutes.

Braised Pigeon¹

**Ingredients**

1 pigeon

28 g butter

½ bay leaf and 1 tsp thyme

121 g onions, sliced

Seasoning (2 shakes of salt and 2 shakes of pepper)

142 ml chicken stock

159 g carrots, diced

14 g flour

80 g turnips, sliced

56 g mushrooms, sliced

Method

1. Melt fat in the pressure cooker and fry onions lightly.

2. Put in the vegetables, herbs, seasoning and stock.

3. Add pigeon and cook for 20 minutes.

4. Stir in flour and bring to the boil.

5. Add mushrooms and cook for 5 minutes.

Pigeon in Cider²

Ingredients

1 pigeon

37 ml chicken stock

½ bacon rasher

¼ bay leaf

30 g onion, shredded

Seasoning (2 shakes of salt and 2 shakes of pepper)

56 g mushrooms, sliced

3 tbsp flour

69 ml cider

#### Method

1. Place pigeon on bacon rasher in a casserole.

2. Cover with onion, mushrooms and stock.

3. Add bay leaf and pour over the cider.

4. Cook, tightly covered, in a slow oven for about 2 hours.

5. Thicken with flour.

Braised Grouse¹

# Ingredients

1 grouse

121 g onions, sliced

90 g celery, sliced

28 g bacon fat

1 tbsp beurre manie

Chicken stock

# Method

# **1. Fry the bird in the bacon fat until brown.**

# **2. Place the onion and celery on the bottom of a casserole and the bird on the vegetables.**

# **3. Pour in enough stock just to cover the bird and cook at 200ºC slowly for 2 hours.**

# **4. Thicken with the beurre manie.**

Grouse Casserole recipe²

**Ingredients**

1 grouse  
50 g butter 
105 g onion, finely chopped 
159 g carrots, finely sliced 
90 g celery, finely sliced  
62 g mushrooms halved with stalks separated from the caps 
1 tbsp red wine vinegar 
50 ml whisky  
2 tsp dried thyme 
1 clove garlic, sliced 
150 ml chicken stock  
Seasoning (2 shakes of salt and 2 shakes of pepper)

**Method**

1. Preheat the oven to 180ºC 
2. Heat half the butter in a frying pan and brown the grouse on all sides. Add the onion, carrot and celery to the pan and then transfer to the casserole. 
3. Melt the remaining butter in the pan and add the mushrooms. Fry for 1 minute and add the wine vinegar and whisky. 
4. Stir and add the herbs, garlic and chicken stock. 
5. Bring to the boil and then pour over the grouse. Season well, then cover and place in the preheated oven for 1 hour.

Partridge a la Crème¹

# Ingredients

# **1 partridge**

# **50 g onion chopped** **10 g butter**

# **250 ml cream** **Season (2 shakes of salt and 2 shakes of pepper)**

# Method

# **1. Fry bird with the onion in the butter until brown.**

# **2. Cook in a covered casserole at 160ºC for 10 minutes.**

# **3. Pour the cream over the bird.**

# **4. Cook for a further 10 minutes, basting frequently.**

Partridge with wine and cinnamon²

# Ingredients

½ tbsp olive oil

1 partridge

1½ garlic cloves

50 ml white wine

1 tsp tomato purée

½ tsp [honey](http://mail.wwt.org.uk/owa/redir.aspx?C=23a32f5032b54374aa89a2a69d427614&URL=http%3A%2F%2Fwww.bbcgoodfood.com%2Fcontent%2Fknowhow%2Fglossary%2Fhoney%2F)

½ tsp concentrated chicken stock

10 [olives](http://mail.wwt.org.uk/owa/redir.aspx?C=23a32f5032b54374aa89a2a69d427614&URL=http%3A%2F%2Fwww.bbcgoodfood.com%2Fcontent%2Fknowhow%2Fglossary%2Folive%2F)

2 shakes of cinnamon

100 g [cherry tomatoes](http://mail.wwt.org.uk/owa/redir.aspx?C=23a32f5032b54374aa89a2a69d427614&URL=http%3A%2F%2Fwww.bbcgoodfood.com%2Fcontent%2Fknowhow%2Fglossary%2Ftomato%2F)

Seasoning (2 shakes of salt and 2 shakes of pepper)

**Method**

1. Heat the oil in a large deep frying pan and quickly brown the partridge all over.

2. Add the garlic and fry until softened.

3. Pour in the wine, bubble over a high heat, then add tomato purée, honey, stock,

olives, cinnamon and seasoning, plus 75 ml of water.

4. Return the birds to the pan, then cover and simmer over a low heat for 20 minutes.

5. Stir in the rest of the ingredients (plus 50 ml of water).

6. Cover and simmer for 20 minutes until tender.

English Roast Duck¹

# Ingredients

# **1 duck** **1 tbsp butter for basting** **2 tbsp plain flour** **275 ml stock** **Seasoning (2 shakes of salt and 2 shakes of pepper)**

## Method

## **1. Heat the butter and baste the duck well. Add stock.**

2. Roast in a fairly hot oven, 190°C, covered with foil, for 1.5 hours, basting

frequently.

3. Sprinkle in the flour and add seasoning.

Duck with Wine²

**Ingredients**

1 duck 
1½ tbsp flour  
1½ tbsp butter 
1½ tbsp olive oil 
¾ tsp salt  
1½ tsp pepper
¼ tsp thyme  
4 shakes dried basil 
150 g onions, chopped  
170 ml white wine 
125 ml cream

**Method**

1. Roll bird in flour and brown in butter and oil, turning often.

2. Add salt, pepper, thyme, basil, onion and wine.

3. Cover with foil and cook at 180ºC for 1 hour.

4. Add cream and cook for 20 minutes.

New England Baked Woodcock¹  
 
**Ingredients**

1 woodcock 
39 mls milk 
1 tbsp flour 
½ tbsp unsalted butter

2 ½ tbsp fine bread crumbs  
½ tsp salt 
½ tsp pepper 
½ tsp paprika 
150 mls single cream

**Method**

1. Dip birds into milk.

2. Roll in flour.

3. Melt butter and brown birds.

4. Dip again into milk.

5. Dredge with bread crumbs.

6. Season with salt, pepper, and paprika.

7. Place in a casserole.

8. Add cream and cover dish.

9. Bake at 180ºC for 45 minutes.

Woodcock a l’orange²

# Ingredients

1 woodcock

20 g croutons

70 ml dry white wine

150 ml concentrated chicken stock

1 orange

2 tbsp flour

# Method

1. Place woodcock on croutons.

2. Add wine, stock, orange zest and juice.

3. Cook for 60 minutes in foil at 180ºC.

4. Add flour to sauce to thicken after cooking.

^1^ Recipe likely to result in approximately neutral pH

^2^ Recipe likely to result in an acidic pH

Recipes were adapted from those found on the following websites:

http://www.timesonline.co.uk/tol/life_and_style/food_and_drink/recipes/

<http://www.cookitsimply.com/>

<http://www.wildliferecipes.net/Game_recipes/game_bird_recipes/>

<http://www.basc.org.uk/en/games-on/topnav/recipes/>

http://www.woodcockminnesota.org/

[http://foodgloriousfood-toto.blogspot.com/](http://mail.wwt.org.uk/owa/redir.aspx?C=23a32f5032b54374aa89a2a69d427614&URL=http%3A%2F%2Ffoodgloriousfood-toto.blogspot.com%2F2007%2F11%2Fpheasant-casserole-with-cider.html)

[http://www.bbcgoodfood.com/recipes/](http://mail.wwt.org.uk/owa/redir.aspx?C=23a32f5032b54374aa89a2a69d427614&URL=http%3A%2F%2Fwww.bbcgoodfood.com%2Frecipes%2F4808%2Fpartridge-with-wine-and-cinnamon)
